# Sestrin 1 ameliorates cardiac hypertrophy *via* autophagy activation

**DOI:** 10.1111/jcmm.13052

**Published:** 2017-02-09

**Authors:** Ruicong Xue, Junyi Zeng, Yili Chen, Cong Chen, Weiping Tan, Jingjing Zhao, Bin Dong, Yu Sun, Yugang Dong, Chen Liu

**Affiliations:** ^1^ Department of CardiologyThe First Affiliated Hospital of Sun Yat‐Sen UniversityGuangzhouChina; ^2^ Key Laboratory on Assisted CirculationMinistry of HealthGuangzhouChina; ^3^ Department of RespiratoryThe First Affiliated Hospital of Sun Yat‐Sen UniversityGuangzhouChina

**Keywords:** Sestrin 1, cardiac hypertrophy, phenylephrine, autophagy, AMPK

## Abstract

Cardiac hypertrophy is one of the major risk factors of cardiovascular morbidity and mortality. Autophagy is acknowledged to be an important mechanism regulating cardiac hypertrophy. Sestrin 1, a downstream target gene of p53, has been proven to regulate autophagy. However, the role of Sestrin 1 in cardiac hypertrophy remains unknown. Our study showed that Sestrin 1 mRNA and protein expression declined in pressure overload cardiac hypertrophy and phenylephrine (PE)‐induced cardiac hypertrophy. Knockdown of Sestrin 1 by RNAi deteriorated PE‐induced cardiac hypertrophy, whereas the overexpression of Sestrin 1 by adenovirus transfection blunted hypertrophy. We discovered that knockdown of Sestrin 1 resulted in impaired autophagy while overexpression of Sestrin 1 resulted in increased autophagy without affecting lysosomal function. In addition, the antihypertrophic effect of Sestrin 1 overexpression was eliminated by autophagy blockade. Importantly, Sestrin 1 targets at the AMPK/mTORC1/autophagy pathway to inhibit cardiac hypertrophy by interaction with AMPK which is responsible for autophagy regulation. Taken together, our data indicate that Sestrin 1 regulates AMPK/mTORC1/autophagy axis to attenuate cardiac hypertrophy.

## Introduction

Cardiac hypertrophy is a responsive mechanism of cardiac overload or damage. The continued presence of hypertrophy deteriorates the malnutrition and death of cardiomyocytes, which results in adverse clinical outcome 1. Cardiac hypertrophy is acknowledged to be one of the predictors of cardiovascular morbidity and mortality 2. At the cellular and molecular levels, cardiac hypertrophy is characterized by the enhancement of cell size, increased protein synthesis and re‐expression of foetal‐type genes such as ANP and BNP. However, the precise mechanism mediating the development and regression of cardiac hypertrophy remains elusive.

Sestrin 1 (also known as PA26), a member of a highly conserved cytoplasmic protein family (Sestrins, consisting of Sestrin 1, 2 and 3), was discovered initially as a target gene of p53 3. Sestrin 1 and 2 were also classified as members of the GADD gene family (growth arrest and DNA damage‐inducible stress–response genes) and are characterized by the ability to regulate cell growth and survivability under various cellular stress conditions 4, 5. Sestrin 1 has been shown to be upregulated by DNA damage and oxidative stress *via* p53 activation and functions as an antioxidant *via* the regeneration of overoxidized peroxiredoxins 6. Moreover, Sestrin 1 was found to participate in mTOR inhibition and thus might share identical mechanisms with mTOR regulation by p53. mTOR has been established as a key inducer of protein synthesis and cell proliferation and as a negative modulator of autophagy 7, 8. Recent evidence has revealed that the loss of *Drosophila* Sestrin resulted in a phenotype of mTOR hyperactivation, which is manifested as defective autophagy, muscle degeneration and cardiac malfunction, and that this effect was prevented by inhibition of mTOR 9. In the cardiovascular system, we previously demonstrated that Sestrin 1 plays a critical role in alleviating angiotensin II‐induced cellular proliferation in neonatal rat cardiac fibroblasts accompanied by mTOR inactivation 10.

Autophagy is a conserved catabolic response to maintaining cellular homoeostasis during stress insults (such as hypoxia and nutrient deprivation) predominantly *via* the lysosomal‐dependent degradation of dysfunctional organelles and misfolded proteins 11, 12. Recent studies have demonstrated that autophagy is involved in a variety of cardiovascular diseases 13. With regard to cardiac hypertrophy, several studies have reported that the downregulation of autophagy was observed during the process of hypertrophy *in vitro* and *vivo* 14, 15. Autophagy is crucial for sustaining myocyte size and cardiac function in the presence of hypertrophic stress. The appropriate activation of autophagy might be conducive to the regression of cardiac hypertrophy. It has been demonstrated that the loss of *Drosophila* Sestrin results in impaired autophagy 9. Thus, considering the potential role of Sestrin 1 in the modulation of autophagy, we examined the regulatory effect of Sestrin 1 in PE‐induced cardiac hypertrophy and explored the underlying mechanisms in this study.

## Materials and methods

### Reagent

PE was purchased from Tokyo Chemical Industry (P0398). The autophagy inhibitor CQ was obtained from Sigma‐Aldrich, St Louis, MO, USA (C6628) and was applied to cardiomyocytes at a concentration of 10 μM 16. Protein G‐Agarose was purchased from Roche (Roche Diagnostics, Mannheim, Germany). For the Western blotting detection of specific proteins, the following primary antibodies obtained from Cell Signaling Technology (Danvers, MA, USA) were used: anti‐Atg7 (#2631), anti‐AMPK (Thr172) (#2531), anti‐AMPK (#2532), antiphosphorylated mTOR (Ser2448) (#5536), antiphosphorylated p70S6K (Thr389) (#9234), anti‐p70S6K (#2708), antiphosphorylated Akt (Ser473) (#4058), anti‐Akt (#9272) and anti‐GAPDH (#2118) antibodies. Anti‐Sestrin 1 antibody was obtained from Abcam, Cambridge, UK (ab134091). Anti‐LC3B antibody was obtained from Novus Biologicals, Littleton, CO, USA (NB100‐2220). Anti‐p62 antibody was purchased from Sigma‐Aldrich (P0068). Antivinculin antibody was purchased from Sigma‐Aldrich (V9264). Anti‐cathepsin D antibody was obtained from Santa Cruz Biotechnology, Dallas, TX, USA (sc‐6486).

### Animals

All of the animal experiments complied with the guide for the care and use of laboratory animals published by the US National Institutes of Health and the Animal Care and Use Committees of the Sun Yat‐sen University. Eight‐ to 10‐week‐old male C57BL/6J mice weighing 24–26 g were anaesthetized through the intraperitoneal injection of 1.5% pentobarbital and underwent descending aortic banding (AB) to induced pressure overload cardiac hypertrophy 17. Four weeks after surgery, the mice underwent with echocardiography and then killed.

### Echocardiography

Four weeks after AB or sham surgery, the mice were administered with inhalant anaesthetics (1.5–2% of isoflurane inhalant mixed with 1 l/min. 100% O2). Transthoracic echocardiograms (Visual Sonics Vevo 2100 with a 30‐MHz transducer) were performed by an experienced technologist blinded to the study group.

### Cardiomyocyte cultures and transfections

Primary neonatal ventricular cardiomyocytes were prepared from 1‐ to 2‐day‐old Sprague Dawley rats. The cardiomyocytes were dispersed by digestion with type I collagenase. The cells were pre‐plated in 100‐mm culture dishes for 1 hr to remove non‐myocytes. The cardiomyocytes were seeded in DMEM/F12 containing 10% FBS and 0.1 mM BrdU at 0.8 × 10^6^ cells per well of a six‐well plate. The knockdown of Sestrin 1 in neonatal cardiomyocytes was achieved by small interfering RNA (siRNA) transfection performed with Lipofectamine^TM^ RNAiMAX according to the manufacturer's instructions. The sequence of siRNA targeting Sestrin 1 was the following: 5` CUGAGAAGGUUCAUGUUAA dTdT 3`. The sequence of siRNA targeting Atg7 was the following: 5′ CCAACACACUUGAGGCUUU dTdT 3′. Thirty‐six hours after seeding, the cardiomyocytes were transfected with Sestrin 1‐specific siRNA (50 nmol/l) and scramble siRNA (50 nmol/l) in serum‐free medium for 12 hrs. After an additional 12 hrs of serum deprivation after transfection, the cardiomyocytes were exposed to hypertrophic stimuli by PE at 50 μM. To overexpress Sestrin 1, a recombinant adenovirus that contained rat Sestrin 1 was constructed by SinoGenoMax Co. Ltd. The recombinant construct contained N‐terminal tagged GFP and C‐terminal tagged His. An adenovirus encoding GFP (Ad‐GFP) was used as a control. Briefly, the cardiomyocytes were transfected with Ad‐GFP‐Sestrin 1‐His (Ad‐Sestrin 1) and Ad‐GFP diluted in medium containing 1% FBS at an MOI of 30 for 6 hrs. After discarding the transfection medium and after 24 hrs of serum‐free medium incubation, the cardiomyocytes were treated with 50 μM PE to induce hypertrophy.

### RNA isolation and quantitative real‐time PCR (q‐PCR)

RNA isolation and q‐PCR were performed as previously described 10. The primers used were as follows: ANP (rat), 5′‐TGAGCCGAGACAGCAAACATC‐3′ (forward) and 5′‐ AGGCCAGGAAGAGGAAGAAGC‐3′ (reverse); BNP (rat), 5′‐CAGCTCTCAAAGGACCAAGG‐3′ (forward) and 5′‐TAAAACAACCTCAGCCCGTC‐3′ (reverse); and GAPDH (rat), 5′‐ACAGCAACAGGGTGGTGGAC‐3′ (forward) and 5′‐TTTGAGGGTGCAGCGAACTT‐3′ (reverse); ANP (mouse), 5′‐ACCTGCACCACCTGGAGG‐3′ (forward) and 5′‐ CCTTGGCTGTTATCTTCGGTACCG‐3′ (reverse); BNP (mouse), 5′‐GCTGTAACGCACTGAAGTTGT‐3′ (forward) and 5′‐ATCACTTCAAAGGTGGTCCCAG‐3′ (reverse); and GAPDH (mouse), 5′‐GTTGTCTCCTGCGACTTCAAC‐3′ (forward) and 5′‐GCTGTAGCCGTATTCATTGTCA‐3′ (reverse).

### Western blotting analysis

The protocol was performed according to a previously reported method 10. The signals were quantified using the software Quantity One (Bio‐Rad, CA, USA).

### Immunofluorescence staining

Immunofluorescent staining was performed as previously described 18. The primary antibodies included rabbit polyclonal anti‐troponin I (1:50, Santa Cruz, sc‐133117), mouse monoclonal anti‐Sestrin 1 (1:50, Santa Cruz, sc‐376170) and rabbit monoclonal anti‐AMPK (1:50, Cell Signaling Technology, #2532), and the immune complexes were detected with Cy3‐conjugated secondary antibodies (1:100, Proteintech Group, Chicago, IL, USA) or FITC‐conjugated secondary antibodies (1:100, Proteintech Group). The nuclei were stained with DAPI (4′,6′‐diamidino‐2‐phenylindole, 0.5 mg/ml, Sigma‐Aldrich, D9542). The images were captured at 400× using an inverted microscope (CKX41; Olympus Corporation, Tokyo, Japan) or at 630× with a confocal microscope (LSM‐710; Carl Zeiss Inc., Oberkochen, Germany).

### Measurement of the cell surface area

Twenty‐four hours after PE treatment, the cardiomyocytes were fixed with 4% paraformaldehyde for 30 min. at room temperature. The relative area of the cardiomyocytes was analysed using the Image‐Pro Plus software (Media Cybernetics, Crofton, MA, USA). Fields of cells with individual treatments were randomly selected, and 50–100 cardiomyocytes in each group were examined in each experiment.

### Evaluation of tandem fluorescent LC3 puncta

Neonatal cardiomyocytes were transfected with adenovirus harbouring mRFP–GFP tandem fluorescent‐tagged LC3 at an MOI of 100 for 6 hrs. Twenty‐four hours after transfection, the cardiomyocytes were exposed to hypertrophic stimuli for an additional 12 hrs. After fixation with 4% paraformaldehyde, images were captured at 630× with a confocal microscope (LSM‐710; Zeiss). For population analysis, at least 20 cardiomyocytes were viewed under a fluorescence microscope (DMI4000B; Leica Microsystems, Hesse, Germany) at 400× magnification.

### Transmission electron microscopy

Briefly, cardiomyocytes were scraped and collected. After fixation by immersion in 2% paraformaldehyde with 2.5% glutaraldehyde for a period of 4 hrs and post‐fixation for 1 hr with 1% OsO4 in 0.1 M phosphate‐buffered saline, the sample was cut into 70‐nm sections using an ultramicrotome, placed on TEM grids, stained with lead citrate and imaged using a Tecnai G2 spirit Twin transmission electron microscope (USA FEI) at 80 kV.

### Co‐immunoprecipitation

Cardiomyocytes lysates were incubated freshly with anti‐AMPK antibody or rabbit normal IgG (negative control) before immunoprecipitation with protein G‐Agarose beads. Treated lysates were immunoblotted with anti‐AMPK and anti‐Sestrin 1 antibodies.

### Statistical analysis

All of the data are expressed as the mean ± S.E.M. from at least three independent experiments. The differences between the means were evaluated using one‐way or two‐way anova followed by Bonferroni post‐test. The statistical significance was established at *P* < 0.05. All of the statistical analyses were performed using the SPSS13.0 software (PASW, SPSS Inc., Chicago, IL, USA).

## Results

### Sestrin 1 expression declines in PE‐ and AB‐induced cardiac hypertrophy

**Figure 1:**
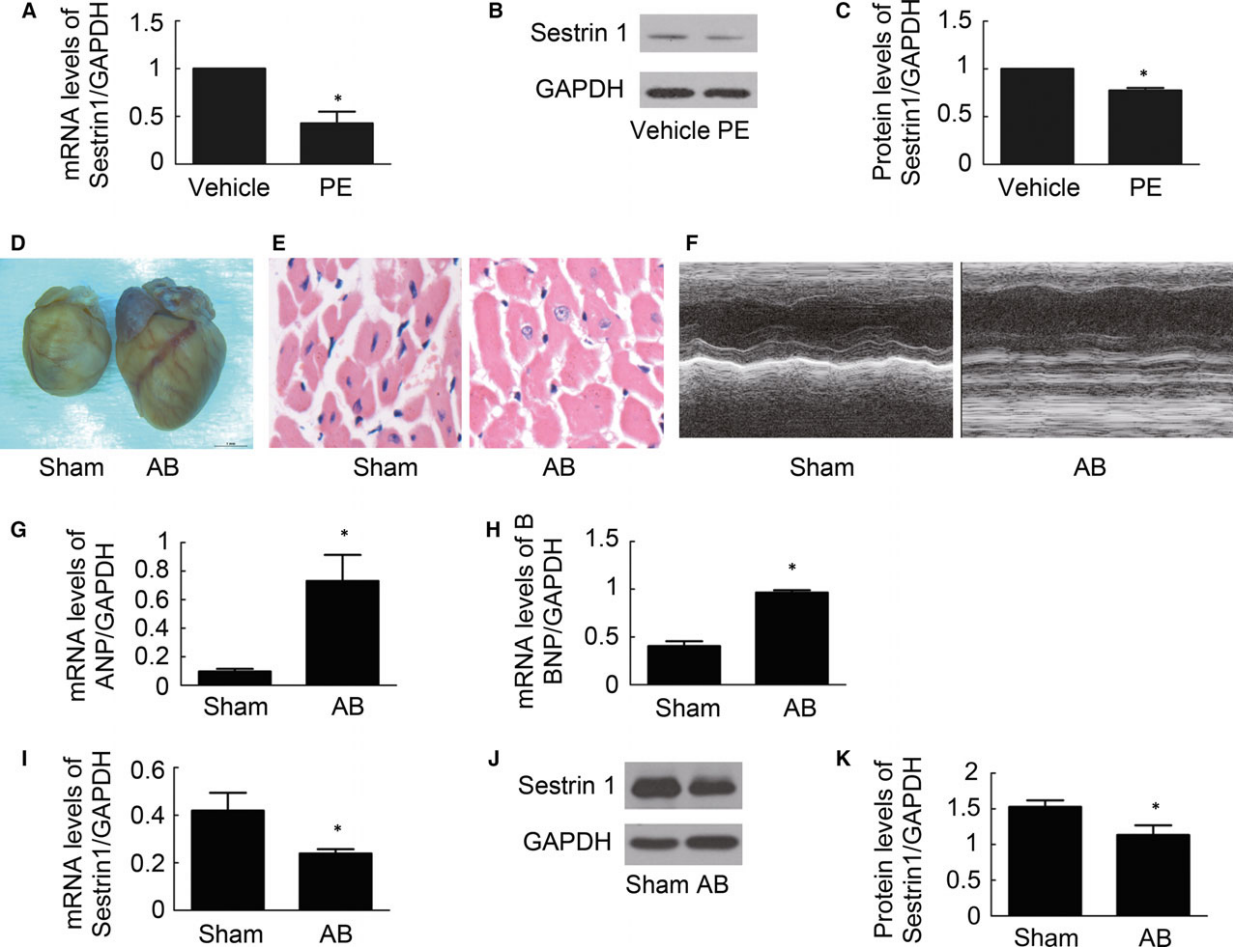
Effect of PE on Sestrin 1 expression in cardiomyocytes. (**A**) The relative mRNA levels of Sestrin 1 mRNA expression 24 hrs after vehicle or PE treatments. (**B**) Representative immunoblots of Sestrin 1 and GAPDH in cardiomyocytes 24 hrs after vehicle or PE treatments. (**C**) Quantitative analysis of data shown in (**B**). (**D, E**) Representative image of the heart and cardiac sections with HE staining from mice 4 weeks after aortic banding or sham surgery. (**F**) M‐mode echocardiography after aortic banding or sham surgery. (**G, H**) The relative mRNA levels of ANP and BNP 4 weeks after aortic banding or sham surgery. (**I**) The relative mRNA levels of Sestrin 1 after aortic banding or sham surgery. (**J**) Representative immunoblots of Sestrin 1 and GAPDH levels in the heart after aortic banding or sham surgery. (**K**) Quantitative analysis of data shown in (**J**). GADPH was used as an internal control. *P < 0.05 *versus* the corresponding vehicle group. The blots represent three independent experiments, *n* = 3–5.

To determine the alteration of Sestrin 1 expression in PE‐induced cardiac hypertrophy, we examined the changes in Sestrin 1 expression 24 hrs after PE treatment. As shown in Figure 1A, compared to the vehicle groups, the mRNA expression of Sestrin 1 markedly decreased 24 hrs after PE incubation. Similarly, the protein expression of Sestrin 1 declined significantly 24 hrs after PE treatment (Fig. 1B,C). The significant alteration in the Sestrin 1 level in PE‐induced cardiomyocyte hypertrophy indicated that this protein played a role in the development of hypertrophy.

To determine whether Sestrin 1 is involved in the regulation of pressure overload cardiac hypertrophy, we detected the alteration of Sestrin 1 expression in aortic banding (AB)‐induced cardiac hypertrophy. Firstly, the hypertrophic model was successfully established and manifested by increased heart weight/body weight ratio (HW/BW), heart weight/tibia length ratio (HW/TL) (Table 1), cardiomyocyte area and mRNA expression of ANP and BNP (Fig. 1D,E,G,H). Moreover, echocardiography data indicated that cardiac function was markedly deteriorated (Table 1 and Fig. 1F). Importantly, we found mRNA and protein levels of Sestrin 1 significantly decreased in cardiac hypertrophy (Fig. 1I,J,K). This alteration indicated that Sestrin 1 might be involved in the regulation of pressure overload cardiac hypertrophy.

**Table 1:** Echocardiographic data showed the phenotype of cardiac hypertrophy induced by AB

Parameter	Sham	AB
Number	*n* = 8	*n* = 9
BW (g)	24.03 ± 1.52	24.72 ± 0.74
HW/BW (mg/g)	5.40 ± 0.34	9.90 ± 1.65*
LW/BW (mg/g)	6.29 ± 0.83	12.61 ± 2.05*
HW/TL (mg/mm)	7.19 ± 0.23	13.47 ± 2.28*
LW/TL (mg/mm)	8.37 ± 0.93	17.18 ± 3.06*
LVEDd (mm)	3.48 ± 0.24	4.28 ± 0.51*
LVEDs (mm)	2.38 ± 0.22	3.20 ± 0.48*
LVPWd (mm)	0.87 ± 0.08	1.23 ± 0.21*
IVSTd (mm)	0.94 ± 0.07	1.26 ± 0.16*
EF%	60.64 ± 3.38	50.30 ± 5.24*

BW, body weight; HW, heart weight; LW, lung weight; TL, tibia length; LVEDd, left ventricular end‐diastolic dimension (diastolic); LVEDs, left ventricular end‐systolic dimension (systolic); LVPWd, left ventricular posterior wall (diastolic); IVSTd, interventricular septum thickness (diastolic); EF, ejection fraction.

Data were represented as mean ± S.E.M. *P < 0.05 *versus* corresponding sham control.

### Sestrin 1 protects against the PE‐induced cardiomyocytes hypertrophic response

**Figure 2:**
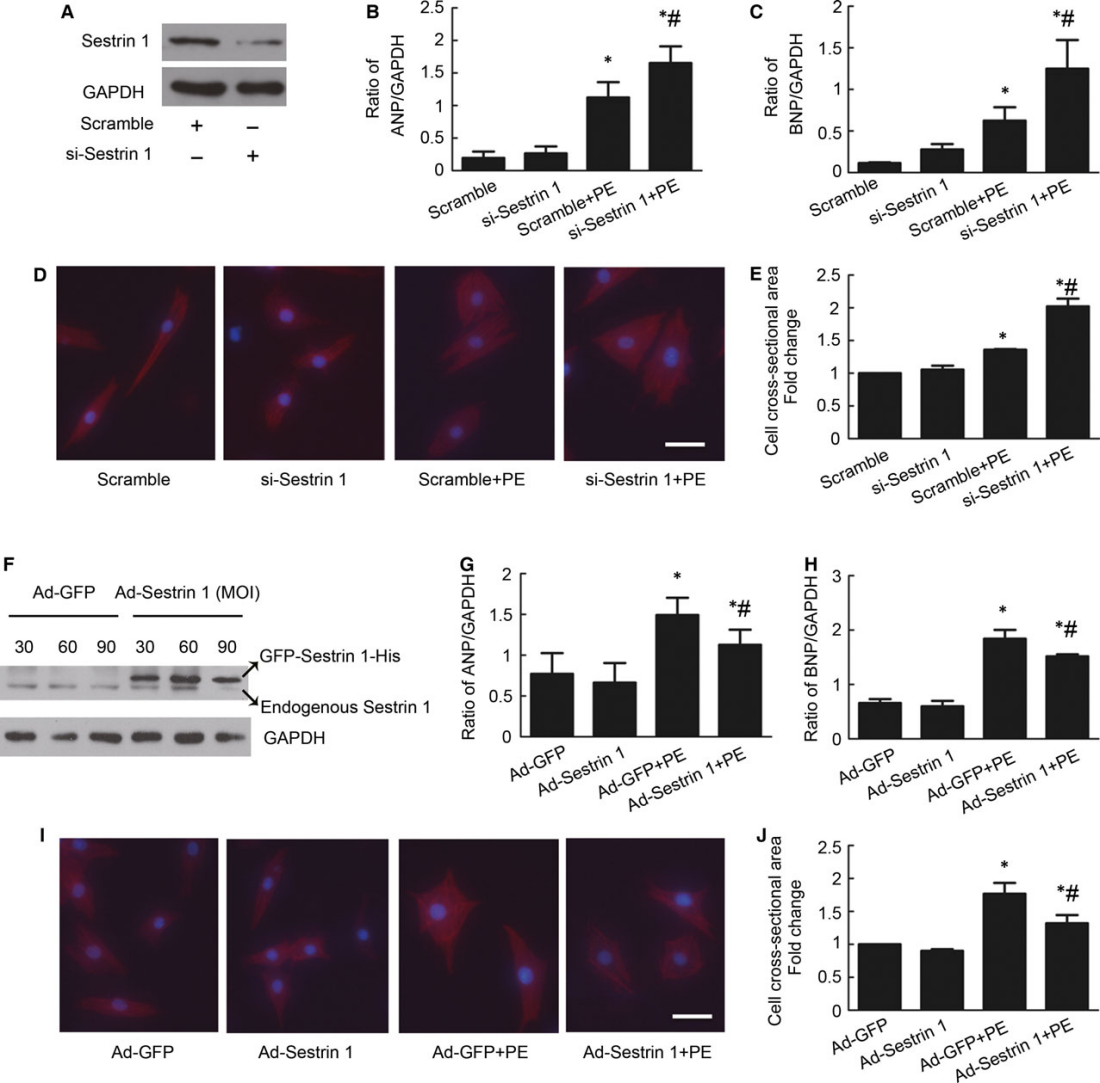
Effect of Sestrin 1 knockdown and overexpression on PE‐induced cardiomyocytes hypertrophy. (**A**) The knockdown effects with siRNA on Sestrin 1 were guaranteed. After siRNA transfection, cardiomyocytes were treated with or without PE for 24 hrs. The relative levels of ANP (**B**) and BNP (**C**) mRNA expression were determined using q‐PCR. (**D**) Cardiomyocytes were stained with troponin I, and the nuclei were stained with DAPI. Scale bar: 50 μm. (**E**) The effect of Sestrin 1 knockdown on the cell surface area after 24 hrs of PE incubation. (**F**) Representative immunoblots of Sestrin 1 after adenoviral transfection with Ad‐GFP or Ad‐GFP‐Sestrin 1‐His (Ad‐Sestrin 1) at different MOIs. After adenoviral transfection, cardiomyocytes were treated with or without PE for 24 hrs. Relative mRNA levels of ANP (**G**) and BNP (**H**) mRNA were measured. (**I**) Cardiomyocytes stained with troponin I. Scale bar: 50 μm. (**J**) The cell surface area was analysed. GADPH was used as an internal control. *P < 0.05 *versus* the corresponding control group without PE, ^#^ *P* < 0.05 *versus* scramble+PE group or Ad‐GFP+PE group. Each of the experiments was repeated three to five times, *n* = 3–5.

To investigate the effect of Sestrin 1 on cardiac hypertrophy, rat neonatal cardiomyocytes were transfected with control siRNA (Scramble) or Sestrin 1‐specific siRNA (si‐Sestrin 1). The inhibitory effect of siRNA on Sestrin 1 protein expression was examined through Western blotting analyses (Fig. 2A) and a knockdown effect of approximately 50% was ensured. Next, we examined the effect of Sestrin 1 knockdown on cardiac hypertrophy by analysing hypertrophic indicators, including ANP, BNP and cardiomyocyte surface area after 24 hrs of incubation with PE. As shown in Figure 2, PE markedly enhanced the mRNA expression levels of ANP and BNP as well as the cell surface area. In the presence of PE, transfection with si‐Sestrin 1 significantly increased the mRNA expression levels of ANP (48.7%) (Fig. 2B) and BNP (86.3%) (Fig. 2C) compared with those observed in the cardiomyocytes transfected with scramble. Similarly, the cell surface area of the cardiomyocytes with Sestrin 1 knockdown was markedly larger compared to that of the cardiomyocytes transfected with scramble (48.8%) (Fig. 2D,E). These data suggested that downregulation of Sestrin 1 deteriorated PE‐induced cardiomyocytes hypertrophy.

To further evaluate the ability of Sestrin 1 to inhibit cardiac hypertrophy, we examined the effect of Sestrin 1 overexpression on cardiac hypertrophy. We achieved the overexpression of Sestrin 1 by transfecting Ad‐Sestrin 1 and used Ad‐GFP as a control. Western blotting analysis confirmed that Sestrin 1 overexpression was approximately 2.6‐fold at an MOI of 30 (Fig. 2F). We next investigated the levels of ANP and BNP as well as the cell surface area in the cardiomyocytes with Sestrin 1 overexpression compared with GFP control under hypertrophic stress. The overexpression of Sestrin 1 abolished the enhancement of the levels of ANP (32.4%) (Fig. 2G) and BNP (21.5%) (Fig. 2H) compared with GFP control in the presence of PE. With regard to the cell surface area, the overall size of the cardiomyocytes transfected with Ad‐Sestrin 1 was significantly decreased (30.0%) (Fig. 2I,J). Therefore, these results solidly verified that Sestrin 1 is a potent inhibitor of PE‐induced cardiac hypertrophy.

### Sestrin 1 activates cardiac autophagy in the process of PE‐induced cardiomyocytes hypertrophy

**Figure 3:**
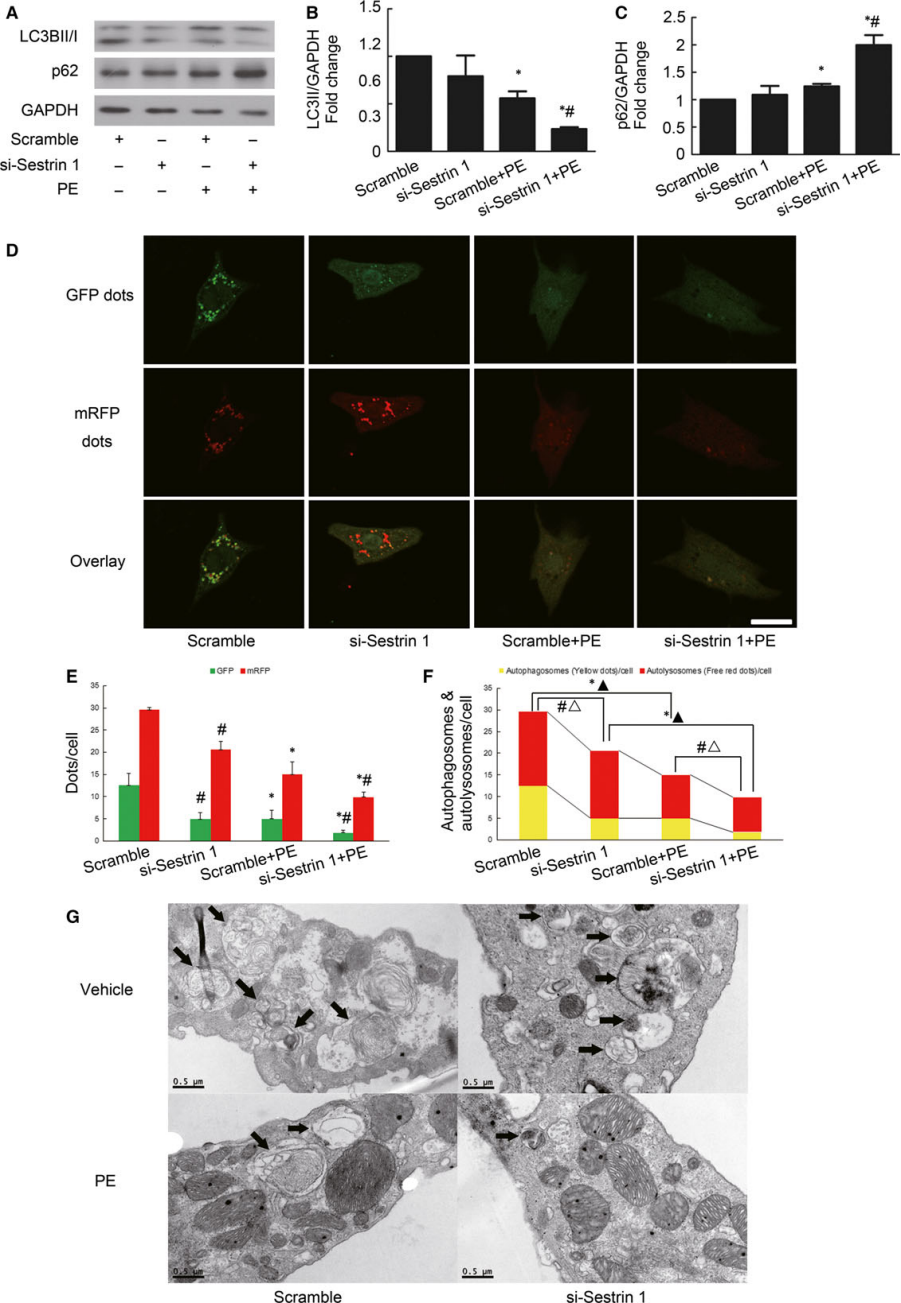
Effect of Sestrin 1 knockdown on autophagy. (**A**) The representative immunoblots and graphs show the changes in the levels of LC3BII (**B**) and p62 after transfection and PE insults. (**C**). GAPDH was used as an internal control. *P < 0.05 *versus* the corresponding control group without PE, ^#^ *P* < 0.05 *versus* scramble+PE group. (**D**) After siRNA transfection, cardiomyocytes were transfected with mRFP–GFP–LC3 adenovirus. Representative images of fluorescent LC3 dots were taken 12 hrs after PE treatment. Scale bar: 50 μm. (**E**) Mean number of GFP and mRFP punctas per cell. *P < 0.05 *versus* the numbers of GFP or RFP dots in corresponding control group without PE. ^#^ *P* < 0.05 *versus* the numbers of GFP or RFP dots in corresponding scramble group. (**F**) Mean numbers of autophagosomes (dots with both red and green fluorescence; yellow fluorescence in the overlaid images) and autolysosomes (dots with only red fluorescence; red fluorescence in the overlaid images). *P < 0.05, ▲*P* < 0.05 *versus* the numbers of autophagosomes and autolysosomes in corresponding control group without PE, respectively. ^#^ *P* < 0.05, ▵*P* < 0.05 *versus* the numbers of autophagosomes and autolysosomes in corresponding scramble group, respectively. The results were obtained from at least three independent experiments. (**G**) Changes in the number of autophagosomes and autolysosomes were shown by transmission electron microscopy (TEM) scanning. Each of the experiments was repeated three to five times, *n* = 3–5.

As a regulatory mechanism of cardiac hypertrophy, autophagy was impaired in our study when the cardiomyocytes were stressed with PE, which resulted in decreased levels of LC3BII (a marker of autophagosome formation) (Fig. 3A,B) and increased levels of p62 (also known as SQSTM, an autophagy adaptor in autophagosome formation and protein turnover, which is degraded by autophagy) 19 (Fig. 3A,C), as well as decreased numbers of autophagosomes and autolysosomes, as assessed through electron microscope scanning which is the gold standard in the evaluation of autophagy (Fig. 3G). Moreover, autophagy was evaluated by transfection with an adenovirus carrying tandem fluorescent mRFP–GFP–LC3 20. When autophagy is activated, LC3 dots are formed. However, mRFP is acid‐resistant whereas GFP loses its fluorescence in the acidic environment of the lysosome when autophagosomes fuse with the lysosome. Thus, the green fluorescent dots indicate autophagosomes while the red fluorescent dots indicate both autophagosomes and autolysosomes. Thus, the yellow dots in the merged images represent autophagosomes. PE significantly reduced the numbers of green and yellow fluorescent dots per cell, indicating impaired autophagy in PE‐induced cardiomyocytes hypertrophy (Fig. 3D,E,F).

To investigate whether Sestrin 1 affects autophagy, we accessed the autophagic indicators in cardiomyocytes with Sestrin 1 knockdown. In the presence of PE, the knockdown of Sestrin 1 resulted in decreased LC3BII (Fig. 3A,B) and increased levels of p62 (Fig. 3A,C), suggestive of impaired autophagy. Moreover, the number of LC3 dots tagged by tandem fluorescent mRFP–GFP in cardiomyocytes with Sestrin 1 knockdown treated with PE was the lowest among all of the groups, suggesting that downregulation of Sestrin 1 further impaired autophagy in PE‐induced hypertrophy (Fig. 3D,E,F). The electron microscope scanning results also indicated that autophagy was blunted in the cardiomyocytes with Sestrin 1 knockdown after hypertrophic stimuli. These results indicated that Sestrin 1 was essential for maintaining autophagy in the process of cardiomyocytes hypertrophy.

**Figure 4:**
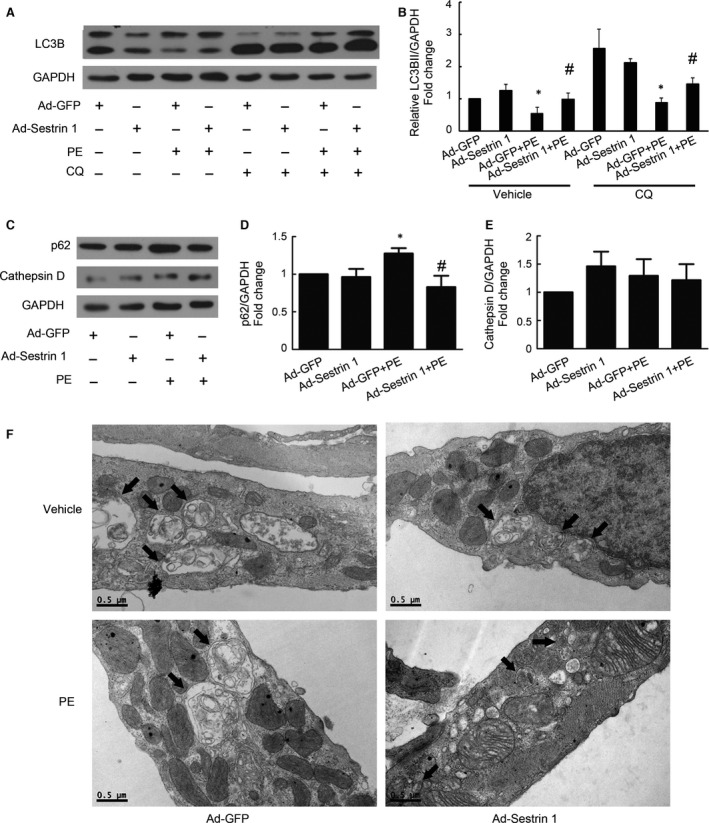
Effect of Sestrin 1 overexpression on autophagy. Cardiomyocytes were treated with or without PE and co‐cultured with or without CQ for 12 hrs. (**A**) The representative immunoblot illustrates the alterations in the levels of LC3BII/GAPDH. (**B**) Quantitative analysis of data shown in (**A**). (**C**) The representative immunoblots illustrates the alterations in the levels of p62 and cathepsin D. Quantitative analysis of levels of p62 (**D**) and cathepsin D (**E**). GAPDH was used as an internal control; *P < 0.05 *versus* the corresponding control group without PE, ^#^ *P* < 0.05 *versus* Ad‐GFP+PE group. (**F**) Ultrastructural images captured using TEM show the changes in the number of autophagosomes and autolysosomes. Each of the experiments was repeated five times, *n* = 5.

Furthermore, we found that in hypertrophied cardiomyocytes, overexpression of Sestrin 1 resulted in significantly increased levels of LC3BII (Fig. 4A,B) and decreased levels of p62 (Fig. 4C,D), indicative of activated autophagy. However, the elevation of LC3BII might be due to increased autophagosome formation or impaired lysosome degradation. Thus, to precisely measure autophagy without interference due to lysosome dysfunction, we assessed the levels of LC3BII in the presence of CQ (chloroquine, an inhibitor of the fusion between autophagosomes and lysosomes) and found enhanced LC3BII levels in the hypertrophied cardiomyocytes with Sestrin 1 overexpression (Fig. 4A,B). We further excluded the disturbance of dysfunctional lysosome degradation in autophagy assessment by measuring the lysosome activity marker cathepsin D. The expression of cathepsin D remained unchanged in cardiomyocytes with Sestrin 1 overexpression (Fig. 4C,E), indicating that Sestrin 1 activates autophagy without disrupting lysosome function. Moreover, using electron microscope scanning, the numbers of autophagosomes and autolysosomes were increased in cardiomyocytes with Sestrin 1 overexpression compared with the GFP control in the presence of PE (Fig. 4F). These results suggested that Sestrin 1 is a positive regulator of autophagy in the process of cardiomyocytes hypertrophy.

### Sestrin 1 ameliorates PE‐induced cardiomyocytes hypertrophy *via* activating autophagy

**Figure 5:**
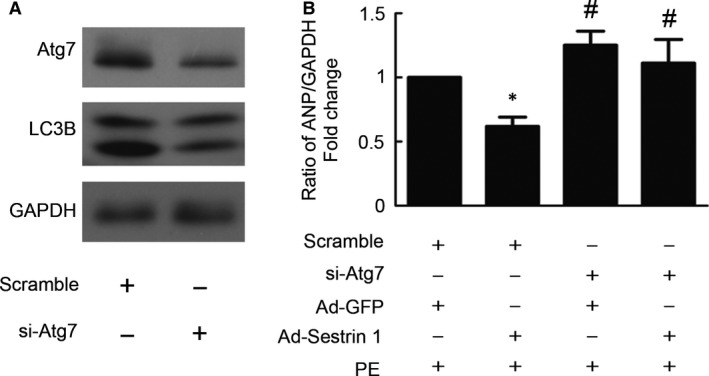
Autophagy blockade weakened antihypertrophic effect of Sestrin 1 overexpression. (**A**) The knockdown effects of siRNA transfection on Atg7 protein expression and autophagy blockade were evaluated. Representative immunoblots of Atg7, LC3BII and GAPDH levels were shown. (**B**) Thirty‐six hours after seeding, the cardiomyocytes were transfected with Atg7‐specific siRNA (50 nmol/l) and scramble siRNA (50 nmol/l) in serum‐free medium for 12 hrs. After an additional 6 hrs of adenoviral transfection with Ad‐Sestrin 1 and Ad‐GFP and serum deprivation, cardiomyocytes were treated with PE for 24 hrs. q‐PCR was performed to determine the relative levels of ANP. GADPH was used as an internal control. *P < 0.05 *versus* scramble+Ad‐GFP+PE group, ^#^ *P* < 0.05 *versus* corresponding scramble+PE group. Each of the experiments was repeated five times, *n* = 5.

To further verify whether Sestrin 1 inhibits cardiac hypertrophy by the activation of autophagy, blockade of autophagy was achieved by siRNA‐mediated knockdown of Atg7 which is essential for autophagy initiation 21. Treatment with Atg7 siRNA transfection significantly reduced the protein level of Atg7 and blunt autophagy, as demonstrated by decreased protein level of LC3II (Fig. 5A). We discovered that in the presence of PE, cardiomyocytes with Atg7 knockdown exhibited a more hypertrophied phenotype than those without. Importantly, we demonstrated that overexpression of Sestrin 1 in cardiomyocytes with Atg7 knockdown did not decrease the levels of ANP as much as those transfected with scramble, suggesting that the protective effect of Sestrin 1 in cardiac hypertrophy was eliminated when autophagy pathway was blunt (Fig. 5B). These findings indicated that the promotion of autophagy is one of the crucial mechanisms involved in the antihypertrophic effect of Sestrin 1.

### Sestrin 1 regulates AMPK/mTORC1 pathway in the process of cardiomyocytes hypertrophy

**Figure 6:**
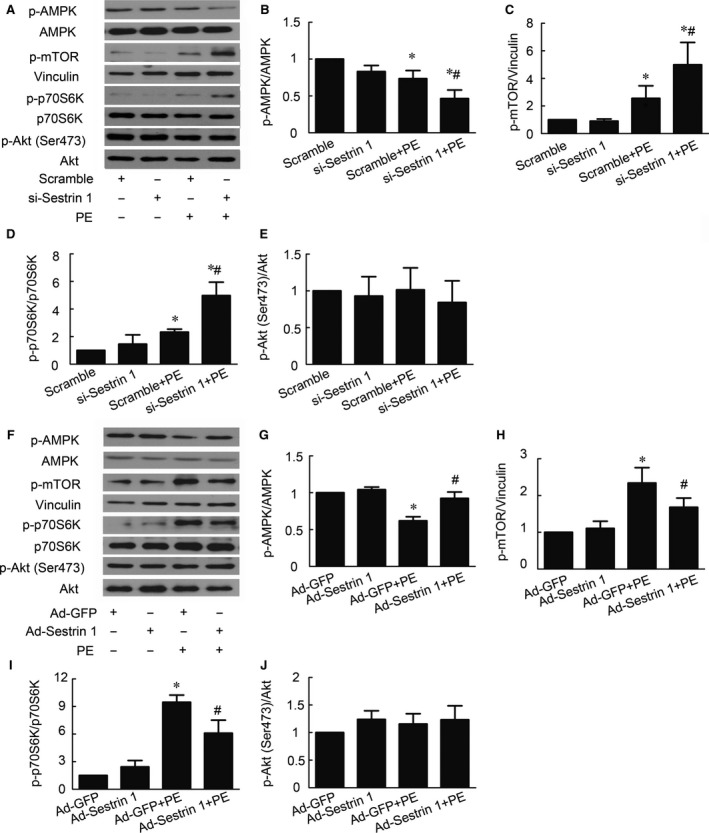
Effect of Sestrin 1 knockdown and overexpression on AMPK/mTOR pathway. (**A**) Representative immunoblots of phosphor‐AMPK, phosphor‐mTOR, phosphor‐p70S6K, phosphor‐Akt (Ser473), AMPK, vinculin, p70S6K and Akt in cardiomyocytes with Sestrin 1 knockdown. (**B**,** C**,** D**,** E**). Quantitative analysis of phosphorylation of AMPK, mTOR, p70S6K and Akt (Ser473) in cardiomyocytes with Sestrin 1 knockdown. (**F**) Representative immunoblots of phosphor‐AMPK, phosphor‐mTOR, phosphor‐p70S6K, phosphor‐Akt (Ser473), AMPK, vinculin, p70S6K and Akt in cardiomyocytes with Sestrin 1 overexpression. (**G**,** H**,** I**,** J**) Quantitative analysis of phosphorylation of AMPK, mTOR, p70S6K and Akt (Ser473) in cardiomyocytes with Sestrin 1 overexpression. *P < 0.05 *versus* the corresponding control group without PE, #*P* < 0.05 *versus* the scramble+PE group or Ad‐GFP+PE group. Each of the experiments was repeated three to seven times, *n* = 3–7.

Furthermore, we aim to illustrate the molecular mechanisms involved in the activation of autophagy by Sestrin 1 in cardiomyocytes hypertrophy. Combining with our previous study illustrating that AMPK promoted autophagy *via* inhibition of mTOR activity 22, we examined whether Sestrin 1 modulates AMPK/mTOR axis. Phosphorylation of AMPK (Thr172), mTOR (Ser2448) and its downstream effectors were examined in cardiomyocytes with Sestrin 1 knockdown or overexpression. After PE stimulation, phosphorylation of AMPK was attenuated while phosphorylation of mTOR was increased. Sestrin 1 knockdown further attenuated the activation of AMPK, thereby increasing the phosphorylation of mTOR (Fig. 6A,B,C); Sestrin 1 overexpression enhanced the activation of AMPK accompanied by decreased phosphorylation of mTOR (Fig. 6F,G,H). We further discovered that in the presence of PE, Sestrin 1 knockdown increased the activation p70S6K, downstream molecule of mTORC1, rather than affecting the activation of AktSer473 (Fig. 6A,D,E), the downstream molecule of mTORC2. Accordingly, Sestrin 1 overexpression resulted in diminished activation of mTORC1 without affecting mTORC2 activation (Fig. 6F,I,J). Combined with the proved regulation on autophagy by AMPK/mTORC1, these findings suggested that the Sestrin 1 target at AMPK/mTORC1/autophagy pathway to inhibit PE‐induced cardiomyocytes hypertrophy.

### Sestrin 1 co‐localizes and interacts with AMPK

**Figure 7:**
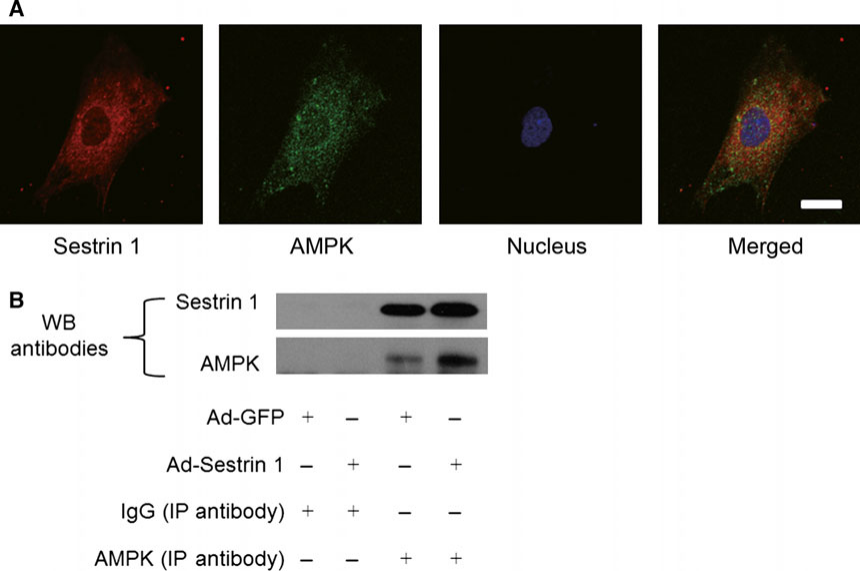
Co‐localization and interaction between Sestrin 1 and AMPK. (**A**) Immunofluorescence microscopy showing co‐localization of Sestrin 1 (red) and AMPK (green) in cardiomyocytes. Scale bar: 50 μm. (**B**) Co‐immunoprecipitation using AMPK antibody or rabbit normal IgG (negative control) in the lysates of cardiomyocytes transfected with Ad‐GFP or Ad‐Sestrin 1. Representative immunoblots with anti‐Sestrin 1 and anti‐AMPK were shown. Each of the experiments was repeated three times, *n* = 3.

Given the modulation of AMPK/mTORC1/autophagy axis by Sestrin 1, we further examined the possible interaction and relationship between Sestrin 1 and AMPK in cardiomyocytes. We assessed the co‐localization of Sestrin 1 and AMPK *via* immunofluorescence, and the images were observed and captured by confocal microscope. In Figure 7A, immunostaining in red indicated the enriched intracellular distribution of Sestrin 1 and immunostaining in green indicated the intracellular distribution of AMPK. The overlaid images presented as yellow indicated that Sestrin 1 co‐localized with AMPK. Furthermore, direct protein–protein interaction between Sestrin 1 and AMPK in cardiomyocytes was confirmed using co‐immunoprecipitation (Fig. 7B). These results indicated Sestrin 1 could modulate activation of AMPK by co‐localization and mutual interaction and thereby exerting its downstream effects to protect against cardiac hypertrophy.

## Discussion

In the present study, we discovered significant decline of Sestrin 1 in AB‐induced pressure overload cardiac hypertrophy and PE‐induced cardiac hypertrophy. Our results demonstrated that the knockdown of Sestrin 1 resulted in deteriorative PE‐induced cardiomyocytes hypertrophy, whereas overexpression of Sestrin 1 protected cardiomyocytes from hypertrophic stress. Moreover, we found that Sestrin 1 antagonized cardiac hypertrophy by positive regulation of autophagy. In addition, we discovered that Sestrin 1 modulated AMPK/mTORC1/autophagy pathway possibly by the co‐localization and mutual interaction with AMPK to exert its antihypertrophic effect.

Previously, we discovered that downregulation of Sestrin 1 in cardiac fibroblasts remarkably increases the generation of ROS and the activation of mTOR, which results in an enhancement of fibroblast proliferation induced by angiotensin II 10. Previous studies that demonstrated the role of Sestrin 1 in the cardiovascular field are limited; how Sestrin 1 functions in cardiac hypertrophy remains unknown. In the present study, we take the lead to study the role of Sestrin 1 in cardiac hypertrophy and the underlying mechanisms. Among all of the pro‐hypertrophic factors, neurohumor factor and pressure overload are the most crucial stimuli involved. Therefore, we study whether Sestrin 1 regulated hypertrophy in different models. *In vivo*, we used aortic banding to induce pressure overload cardiac hypertrophy. We discovered that as the progression of cardiac hypertrophy and deterioration of cardiac function, Sestrin 1 mRNA and protein expression significantly declined. *In vitro*, we discovered that Sestrin 1 mRNA and protein levels were significantly decreased after PE treatment. Taken together, our data strongly indicated that Sestrin 1 regulated both pressure overload cardiac hypertrophy and PE‐induced cardiac hypertrophy. Combined with previous studies demonstrating that Sestrin 1 expression is upregulated under cellular stress 4, 5, our findings suggested that Sestrin 1 might play a protective role under hypertrophic stress and is depleted when the stress is persistent.

To further confirm the possible effect of Sestrin 1 in cardiac hypertrophy, we investigated the ‘gain and loss’ phenotype of Sestrin 1 in PE‐triggered cardiomyocytes hypertrophy. Cardiomyocytes with Sestrin 1 knockdown exhibited a more exacerbated hypertrophic phenotype, manifested as increased levels of foetal‐type genes (ANP and BNP) and increased cell surface area. Conversely, the adenoviral overexpression of Sestrin 1 significantly inhibited cardiac hypertrophy. Based on the phenotype analysis, we found that Sestrin 1 is a potent antihypertrophic effector.

Autophagy manifests as the formation of double‐membrane vesicles that fuses with the lysosome to eliminate dysfunctional proteins and organelles 23, 24. Under basal conditions, autophagy is essential to maintaining cellular homoeostatic control, whereas excessive autophagy results in cell death and apoptosis 25. Aberrant autophagy is unfavourable for normal tissue and organismal function and leads to various diseases such as congenital myopathies 26 and Parkinson disease 27. Cardiac hypertrophy is regulated by autophagy in response to hypertrophic stress, such as pressure overload and neurohumoural stimuli 28. The appropriate activation of autophagy during cardiac stress is crucial to maintaining cellular homoeostasis. Chen *et al*. reported that autophagy promoted by intermedin protects cardiomyocytes from hypertrophic stress 29. *In vivo* evidence demonstrates that cardiac‐specific Atg5‐deficient mice develop cardiac dysfunction and left ventricular dilatation 1 week after transverse aortic constriction 14. These studies illustrate that autophagy is an adaptive mechanism employed to resist hypertrophic stress potentially *via* the promotion of protein turnover to avoid damaged proteins and organelles. In our study, various autophagy indicators were found to be markedly reduced after PE insult and autophagy blockade by Atg7 knockdown resulted in a more hypertrophied phenotype. Our results indicated that a loss of autophagy might present a deteriorating effect in the process of cardiac hypertrophy. Previous reports have revealed that Sestrin 1 is involved in regulating autophagy, as demonstrated by the impaired autophagy due to the loss of *Drosophila* Sestrin. Consistent with the previous results, our results showed that Sestrin 1 knockdown significantly impaired autophagy, suggesting that Sestrin 1 expression is essential for maintaining autophagy in the process of cardiac hypertrophy. Furthermore, the evidence that overexpression of Sestrin 1 activated autophagy in the presence of PE directly proved that Sestrin 1 is a positive activator of autophagy in cardiac hypertrophy. Importantly, autophagy blockade by Atg7 knockdown partly retarded the antihypertrophic effect of Sestrin 1, indicating that activation of autophagy plays a pivotal role in the protection against hypertrophic stress mediated by Sestrin 1.

AMP‐activated protein kinase (AMPK) is a critical sensor of the cellular energy status, activation of which has been proved to promote autophagy 30. We previously demonstrated that activation of AMPK by either pharmacological stimulation or adenovirus transfection significantly inhibited cardiac hypertrophy *in vitro* and *in vivo*; its positive regulation on autophagy *via* the inhibition of mTORC1 was one of the mechanisms involved 22. Downstream of AMPK, mTOR is a conserved regulator that governs the aspects of cell function including autophagy, cell size, transcription and translation 31, 32, 33. Depending on its binding partners, mTOR consists of two different kinase complexes, mTORC1 and mTORC2. mTORC1 mainly regulates the initial stages of autophagy. A series of molecules involved in the induction stage of autophagy such as ULK1 and PP2A are directly modulated by mTORC1 34, 35. A study demonstrated that mTORC1 also regulated the formation and fusion of the autophagic compartment with lysosomes 36. However, the role of mTORC2 in regulating autophagy remains debated 37, 38. We have previously illustrated that AMPK/mTORC1 axis is a key regulatory signalling pathway in cardiac hypertrophy with the involvement of autophagy 22. Consistent with our previous study, we discovered that PE significantly reduced the phosphorylation of AMPK and thereby inducing the phosphorylation of mTORC1, an effect that directly results in inhibited autophagy. It was reported that Sestrin 1 is one of the newly found genes that links p53 and AMPK/mTOR pathway; under stress, Sestrin 1 inhibits mTOR by activation of AMPK 39. To further clarify the molecular mechanisms involved in the regulation of autophagy in cardiac hypertrophy by Sestrin 1, we examined the effect of Sestrin 1 on AMPK/mTOR pathway. Importantly, Sestrin 1 markedly enhanced the activation of AMPK and depressed the activation of mTORC1 under PE insults, which initiate the revival of autophagy. Combining with our previous study demonstrating that activated AMPK inhibits cardiac hypertrophy *via* promoting autophagy, our current study further indicated that Sestrin 1 modulates AMPK/mTORC1/autophagy axis to fulfil its antihypertrophic function.

It was discovered that Sestrin 1 directly activates AMPK at Thr172 *via* the mutual interaction; however, this interaction has not been confirmed in cardiomyocytes. We discovered that the intracellular distribution of AMPK and Sestrin 1 overlaid, suggestive of their co‐localization in cardiomyocytes. By the utilization of Co‐IP, the mutual interaction between Sestrin 1 and AMPK in cardiomyocytes was further verified. Based on our results, co‐localization and interaction between Sestrin 1 and AMPK might be the core mechanism employed for the regulation of AMPK/mTORC1/autophagy axis by Sestrin 1.

In summary, our study revealed that Sestrin 1 ameliorates both PE‐ and AB‐induced cardiac hypertrophy; its positive regulation on autophagy by targeting AMPK/mTORC1 axis is one of the critical mechanisms involved. Our demonstrations shed light on the role of autophagy induction in therapeutic strategy and could provide a potential new target gene for pathological cardiac hypertrophy.

## Conflict of interest statement

none.
